# Lettuce Production in Intelligent Greenhouses—3D Imaging and Computer Vision for Plant Spacing Decisions

**DOI:** 10.3390/s23062929

**Published:** 2023-03-08

**Authors:** Anna Selini Petropoulou, Bart van Marrewijk, Feije de Zwart, Anne Elings, Monique Bijlaard, Tim van Daalen, Guido Jansen, Silke Hemming

**Affiliations:** Business Unit Greenhouse Horticulture, Wageningen University & Research (WUR), 6708 PB Wageningen, The Netherlands

**Keywords:** artificial intelligence, computer vision, sensors, lettuce, indoor farming, autonomous greenhouses, climate control, plant spacing, remote control, data driven growing

## Abstract

Recent studies indicate that food demand will increase by 35–56% over the period 2010–2050 due to population increase, economic development, and urbanization. Greenhouse systems allow for the sustainable intensification of food production with demonstrated high crop production per cultivation area. Breakthroughs in resource-efficient fresh food production merging horticultural and AI expertise take place with the international competition “Autonomous Greenhouse Challenge”. This paper describes and analyzes the results of the third edition of this competition. The competition’s goal is the realization of the highest net profit in fully autonomous lettuce production. Two cultivation cycles were conducted in six high-tech greenhouse compartments with operational greenhouse decision-making realized at a distance and individually by algorithms of international participating teams. Algorithms were developed based on time series sensor data of the greenhouse climate and crop images. High crop yield and quality, short growing cycles, and low use of resources such as energy for heating, electricity for artificial light, and CO_2_ were decisive in realizing the competition’s goal. The results highlight the importance of plant spacing and the moment of harvest decisions in promoting high crop growth rates while optimizing greenhouse occupation and resource use. In this paper, images taken with depth cameras (RealSense) for each greenhouse were used by computer vision algorithms (Deepabv3+ implemented in detectron2 v0.6) in deciding optimum plant spacing and the moment of harvest. The resulting plant height and coverage could be accurately estimated with an R^2^ of 0.976, and a mIoU of 98.2, respectively. These two traits were used to develop a light loss and harvest indicator to support remote decision-making. The light loss indicator could be used as a decision tool for timely spacing. Several traits were combined for the harvest indicator, ultimately resulting in a fresh weight estimation with a mean absolute error of 22 g. The proposed non-invasively estimated indicators presented in this article are promising traits to be used towards full autonomation of a dynamic commercial lettuce growing environment. Computer vision algorithms act as a catalyst in remote and non-invasive sensing of crop parameters, decisive for automated, objective, standardized, and data-driven decision making. However, spectral indexes describing lettuces growth and larger datasets than the currently accessible are crucial to address existing shortcomings between academic and industrial production systems that have been encountered in this work.

## 1. Introduction

Recent studies strongly indicate that food demand will increase by 35–56% over the period of 2010–2050 as a result of population increase, economic development, and urbanization, among other drivers [1]. The expected increase in food demand places pressure on natural resources and may lead to negative environmental impacts as well as biodiversity losses [2]. Among the possible solutions are the transformation of food production into a green industrial process and the promotion of policies for plant-based and high-nutrient diets [3].

Greenhouse systems allow sustainable intensification of food production with demonstrated high crop production per cultivation area [4]. While vegetable production is increasing in area and volume, the number of farms declines, resulting in more vegetable area and volume per farm and per grower [5]. At the same time, the availability of labor is an industry-wide challenge as well as the lack of experienced managers and growers in crop production. Greenhouses are highly dynamic production systems operating through an integrated set of activities performed by growers [6]. Growers need to consider various performance indicators such as yield, quality, timing, and sustainability standards and meet the volatile market demands, and prices in uncertain environmental conditions subject to weather conditions, for example [7].

Modern horticultural production is highly dependent on up-to-date information on farm operations. Production processes are already highly automated and controlled [8]. Information systems driven by the rapid developments in cloud computing, the Internet of Things, Big Data, machine learning, augmented reality, and robotics are changing the horticulture horizon toward precision horticulture [9,10,11,12]. Digital technologies, computational power, and high-fidelity sensors act as catalysts in the transition toward advanced and autonomous production systems. Non-invasive, near real-time data and information with high spatial and temporal resolution create opportunities for advisory or automated decision software and the design of advanced models, known as digital twins [13]. Monitoring and interpretation of the system’s dynamics at coarser and granular levels allow for location-specific operations to ascertain desired conditions that meet crop demands.

Digital twins are equivalent to real-life objects mimicking the behavior and states over their lifetime in virtual space [13]. Greenhouse digital twins can be seen as coupled dynamic climate and crop models representing the actual physical, biological, and integrated technical systems as virtual representations of reality [14]. Digital twins can be used to simulate the effects of different growing conditions and crop management strategies, give insights into their effect on performance indicators, and support decision-making [15]. There have been several achievements in the implementation of mechanistic crop and climate models in horticultural research to facilitate decision making in greenhouse operations [16,17,18]. Broadly validated dynamic models of the greenhouse climate and crop include, e.g., KASPRO [19] and INTKAM [20], which have been used for several research activities. A benchmark experiment in optimizing net profit using AI for the remote control of cucumber cultivation in 2018 [21] and a follow-up on optimizing the net profit of tomatoes as a function of yield and quality a year later [22], showed the potential of AI in controlling and outperforming human decisions by experienced growers. Automated greenhouse control wasthus demonstrated to be possible; therefore, our next focus was on the autonomy, robustness, and scalability of such control systems [23]. The goal of the third edition of the Autonomous Greenhouse Challenge was the full autonomous control of lettuce cultivation.

Commercial greenhouse production of lettuce (*Lactuca sativa* L.) is already highly automated. Lettuce is grown in controlled greenhouse environments including hydroponic, aquaponic, and vertical growing systems. The systems minimize labor requirements by using conveyor belts and lifts throughout the growing processes from seedling to harvesting [24]. Lettuce hydroponic systems include Nutrient Film Techniques (NFT), Deep Flow Techniques (DFT), as well as Ebb and Flow systems. NFTs are the most widespread method of recirculating nutrient solution systems [25] and employ a shallow stream of water with dissolved nutrients flowing over the roots of plants in water-tight gullies, here referred to as gutters. The nutrient solution is initially stored in a reservoir, pumped out into the gutters at an angle, and drained to a tank for filtering before re-cycling to the reservoir for re-use. Gutters are automatically filled with the growing media and lettuce heads and transported on conveyor belts to the main greenhouse area. When lettuce heads are fully grown, they are moved toward the harvesting area. At the harvesting area, cutting machines remove the plants from the gutters and transfer the lettuce heads for packaging while the gutters are washed, and the process starts again. During the growing period in the greenhouse, the distance between the gutters and crops on the gutters largely determines the required amount of greenhouse space and, therefore, resource use. From the perspective of greenhouse automation, it is important to note that the automated optimization of lettuce plant spacing is not yet implemented in practice.

Optimal cultivation temperatures for lettuce are relatively low and range from 15.5 °C to 28 °C during the daytime to 3 °C to 12 °C at night time [26]. The optimal pH ranges for the nutrient solution from 5.8 to 6.5 and its optimal electrical conductivity (EC) should be 1.5 mS/cm [27]. A wide variety of crop types can be distinguished among the existing lettuce cultivars, with crisp head and butterhead commonly grown in the United States and Western Europe, respectively, whereas Romaine and loose-leaf types are mainly cultivated in Mediterranean areas [28]. The crop is susceptible to physiological problems including outer leaf tip burn, inner tip burn, and discoloration of ribs [29]. Growth of lettuces, as with any crop, is related to incident radiation and CO_2_ concentration, and due to the relatively high surface area to volume ratio, has high transpiration rates [29]. A fully autonomous decision of optimum climate setpoints can contribute to better crop growth and lower resource use.

Since plant spacing is an important criterion for good vegetative growth on an m^2^ basis, it is a major aspect of yield maximization. Densely planted lettuces can obstruct morphological characteristics such as head size, leaf expansion and color, and compactness [30,31,32]. Wider spacing ensures higher light availability per head and that nutritional requirements are satisfied; however, this comes at the expense of less efficient utilization of the growing area and resources used. Optimum plant spacing is a management decision in hydroponic lettuce cultivation that can potentially be determined using 3D camera images and other sensor data, together with artificial intelligence algorithms to fully automate the operational process.

Modern camera systems and innovative artificial intelligence (AI) technologies such as computer vision allow objective, non-invasive, and continuous data for precision horticulture applications [33]. Advances in machine learning for image processing have resulted in a wide range of research and applications for crop monitoring [34]. Applications of computer vision can be found in the fields of pests, disease or weed detection [35,36,37], fruit and flower detection, counting and fruit ripeness [38,39], crop stress detection [40], yield estimation, or moment of harvesting [41,42]. Moving cameras or flying drones with mounted cameras scan plants from various viewpoints, addressing matters of occlusion and creating 3D representations of the crop [43]. High-resolution imaging in combination with deep learning techniques is expected to have great potential for precision farming and remote control operations for purposes of autonomous greenhouses [44].

Traditional computer vision techniques struggle with the challenging greenhouses environment because of varying environmental conditions. Light conditions are continuously changing, and occlusion makes it difficult to identify individual plants or plant organs [45]. The development of hand-crafted algorithms was often time-consuming and not reliable enough. However, recent development in the field of deep learning made it easier to use vision systems in greenhouses. High classification accuracies of up to 99.7% [46] on large plant datasets such as the “Oxford-Flowers102” [47] dataset show the power of deep learning for plant phenotyping. Already in 2017, the first paper appeared on the quality assessment of lettuce using artificial neural networks [48]. Lettuce was binarily classified as good” or “reject”. Although the algorithm was not complex as it had only two layers, it was one of the first publications that showed the possibility of using neural networks for lettuce classification. The ability of networks to learn plant features from single lettuce images can be determined by the recently published lettuce dataset [49]. At the moment, three papers have been published, obtaining high accuracy to estimate fresh weight from the images with a Root Mean Squared Error (RMSE) up to 25.3 g [50,51,52].

The above-mentioned examples are only focusing single lettuce images. With the development of instance segmentation algorithms it is possible to determine the growth rate of lettuce over time by extracting the leaf area of single lettuce plants, as seen in [53], their experiments were in a semi-commercial setting without overlapping lettuces. A more commercial example can be found in [54], in which aerial images were collected and the number of lettuces was determined including a size estimation into three different categories. One of the conclusions was that despite the fact that many individual lettuces can be detected, there is still a gap between object detection and trait measurements [54]. In greenhouses, the environmental conditions are much better for high-quality imaging, reducing the AI and trait measurement gap. Other researchers developed a high-throughput system for individual plant phenotyping of lettuce [55]. Each lettuce head was placed in an individual pot; by detecting the pot and by applying semantic segmentation, many plant traits were calculated including projected area and perimeter. The area and size are two of the most interesting growth indicators. However, when the leaves became larger than the pot size, prediction accuracy decreased; as a result, the growth curves were only accurate for the first weeks. It can be noticed that most experiments were carried out in semi-commercial conditions. When the leaves were overlapping, either the experiment stopped, or the extraction of the parameters was removed. Next to that, in each experiment, the interpretability of the results was difficult. There is still a mismatch between object detection, determining plant traits, and more importantly, what a grower should do with the provided information. If AI can extract growth rate, how should a grower use this information to improve the cultivation? Therefore, more advanced methods are needed that can extract information in greenhouses and conform to commercial practice while maintaining interpretability.

This paper describes the results of the third Autonomous Greenhouse Challenge, an experiment in which the autonomous control of lettuce production has been realized in six different greenhouse compartments, each controlled by AI algorithms developed by participating teams. During the experiment, the goal was to decide upon climate and crop management strategies to optimize the net profit of lettuce production, considering yield and product prices, resource use, and costs including greenhouse occupation. The experiment provided valuable public datasets which can be used for future AI training purposes, and which can be found under the Data Availability Statement. In this paper, we give an overall analysis of the results obtained by the teams. Next to that, we focus on the research question, of how computer vision and deep learning algorithms can be used for automated operational decisions of lettuce greenhouse production, as currently plant spacing and harvesting are determined on fixed schedules since transplanting. Furthermore, we examine how better utilization of the occupied growing area, efficient resource use that meets crop growing demands, and timely planning of harvest events can be supported by non-invasively estimated indicators such as the proposed light loss and harvest indicator. Results of other studies focus on answering similar questions on crop trait detection with computer vision in highly controlled and steady environmental conditions. This research realizes steps closer to commercial practice by processing smaller datasets of canopy images under varying environmental conditions.

## 2. Materials and Methods

**Figure 1:**
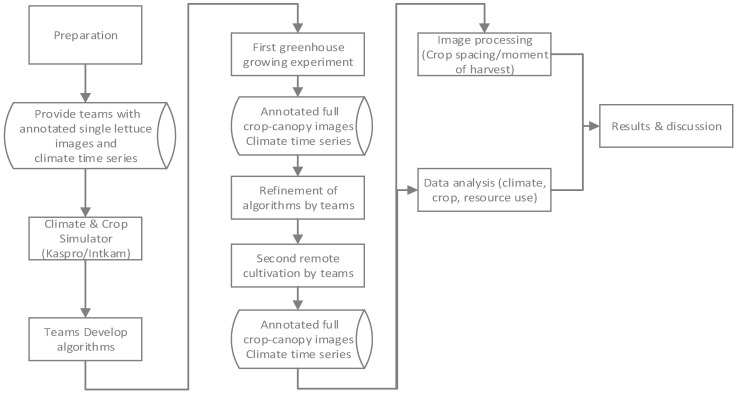
Research methodology of the growing experiments and analysis of results. Data from the annotated single lettuce images is found in [49], whereas the annotated full crop data are found in [56].

This paper describes different steps of the realized research methodology, and an overview is given in Figure 1. In the preparation phase teams developed their own AI algorithms based on provided annotated single lettuce images and climate data time series from a climate and crop simulator [49]. After this preparation phase, two lettuce-growing experiments were conducted in greenhouses at Wageningen University & Research (Section 2.1. Greenhouse compartments and equipment Section 2.2. Crop and Section 2.3 Greenhouse climate and crop control). During the first greenhouse experiment, teams could gain experience in controlling the lettuce growth based on real-time data from the greenhouse (Section 2.4 Data communication, Section 2.5 Remote sensing, and data collection). New annotated images, of the full crop under the camera’s field of view and climate time series data were collected [56]. Teams could refine their algorithms before the second greenhouse experiment. Another set of annotated images, full crop canopy images, and climate times series were collected [56]. After the experiments, an analysis of climate, crop, and resource use was made and given in this paper (Section 3. Results Section 3.1, Section 3.2 and Section 3.3). An additional analysis of plant spacing decisions was made (Section 3. Results Section 3.4) based on different image processing methods (Section 2.6 Image processing for plant spacing decisions). The results are discussed and concluded in Section 4 and Section 5.

### 2.1. Greenhouse Compartments and Equipment

Each greenhouse compartment at the research facility of Wageningen University & Research in Bleiswijk, The Netherlands, had a size of 96 m^2^. The compartments were equipped with standard actuators also available in commercial high-tech greenhouses as shown in Figure 1. A pipe-rail heating on the floor with a peak capacity of 120 W/m^2^ controlled by the heating temperature setpoints, continuous roof ventilation (ventilation area of 0.3 m^2^ opening per m^2^ greenhouse area, equipped with anti-thrips netting), two types of inside moveable screens (LUXOUS 1547 D FR energy screen and OBSCURA 9950 FR W light blocking screen, Ludvig Svensson), white LED artificial lights of dimmable intensity controlled in a continuous range between 27 and 270 µmol/m^2^/s and efficiency of 2.4 μmol/J, (VYPR 2p, Fluence by Osram), a fogging system (maximum capacity of 330 g/m^2^/h), and CO_2_ supply (maximum capacity 15 g/m^2^/h) were available. Plants were grown in soil-pressed pots on NFT hydroponic gutters (Hortiplan, Belgium) placed on an inclination. A recirculating water system was supplying water and nutrients via pressure-compensated narrow tubes injecting water into the gutters.

The experiment of the third Autonomous Greenhouse Challenge was conducted in the first half of 2022 in six different high-tech Venlo-type greenhouses compartments of Wageningen University & Research, in Bleiswijk, The Netherlands. The basic greenhouse construction and equipment with actuators as well as the standard sensors and control of the greenhouse compartments were identical to the elements which can be found in commercial greenhouses (see Section 2.2 Crop and Section 2.3 Greenhouse climate and crop control). However, the greenhouse compartment size was much smaller than in commercial practice. Different teams (CVA, DigitalCucumbers, Koala, MondayLettuce, VeggieMight) and a reference were controlling the six compartments.

### 2.2. Crop

Two cultivation cycles of lettuce cv. “Lugano” (Rijk Zwaan, The Netherlands) were conducted in 6 equal greenhouse growing compartments. Lettuces were grown in a hydroponic NFT system. Seeds were propagated to seedlings 8 weeks before the transplanting date. The young plants were grown in cubes of compacted peat. On the days of transplanting (2 February and 3 May 2022, respectively), the seedlings were placed in the greenhouse compartments in small holes of slightly tilted gutters to which water with nutrients was supplied at a certain frequency.

The lettuces were grown in 3.2 m plastic gutters, all having 30 plant holes, with an 11 cm heart-to-heart distance. The gutters were 10 cm wide, so the maximum plant density was 92 (rounded) plants per m^2^ in the initial stage. Lettuces were grown in two rows of such gutters as depicted in Figure 1. Plant appearance, pests, and diseases were monitored weekly by experts without interfering with any operational control decisions in the compartments. Irrigation and nutrient recipes were determined by the experienced greenhouse staff of the Bleiswijk Research Center.

**Figure 2:**
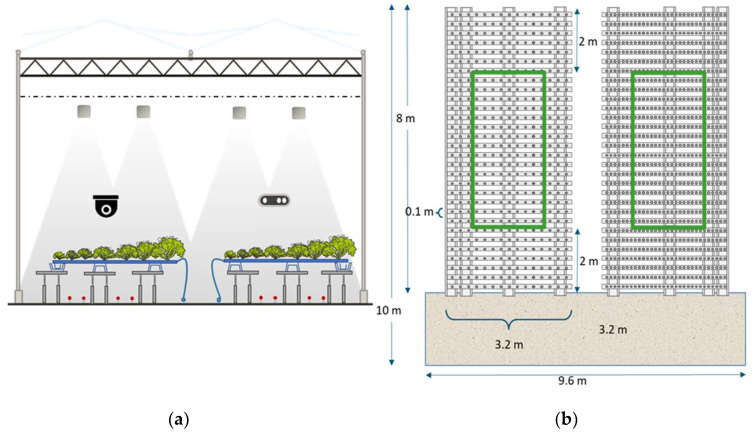
(**a**) Cross- and (**b**) top-view sections of one greenhouse experimental compartment with 96 m^2^ ground floor. (**a**) Compartment with crop and actuators: rail pipe, irrigation system, NFT gutters, CO_2_ supply, LED artificial light, and two screens. (**b**) Arrangement of lettuce gutters. Green boxes represent the harvest area for data analysis.

Leafy vegetables are sellable to retail at a particular weight and shape. The lettuce heads in the area of evaluation (Figure 2) were classified at the moment of harvest into three categories. Class A were sellable lettuces with a minimum average weight of 250 g, Class B were lettuces with a weight between 220 and 250 g, and Class C were non-sellable lettuce heads that were underweighted and or showed visible deformations. Malformations referred to quality aspects related to the shape of the plant and defects of the leaves (e.g., leaf discoloration, leaf rotting, and diseases).

### 2.3. Greenhouse Climate and Crop Control

Strategic and operational climate control was carried out by participating teams of the third Autonomous Greenhouse Challenge. Strategic decisions include, e.g., the use (installation) of screens or artificial lighting or the starting density of the crops, operational decisions included, e.g., the timing and amount of screen or lighting hours or crop spacing decisions. The AI algorithms of the teams were determining control setpoints of the heating temperature, CO_2_ concentration, humidity deficit, lighting intensity, operation of the blackout, and energy screens, as well as leeward and windward ventilation. The mechanistic climate and lettuce crop models of WUR (KASPRO and INTKAM, respectively) could be used by the teams as a training environment for the algorithms before the start of each cultivation cycle.

Resource use was calculated based on measured data: heating energy (MJ/m^2^) with a price of 0.0375 EUR/kWh, electricity (kWh/m^2^) with a price of 0.125 EUR/kWh for the on-peak hours (07:00–23:00), and 0.075 EUR/kWh for the off-peak hours, CO_2_ use (kg CO_2_/m^2^) with a price of 0.12 EUR/kg.

As in commercial practice, the spacing system allows for several plant densities; densities could be reduced from the starting density of 92 heads per m^2^ via a density of 60, 45, 30, 23, and 18, to the lowest density of 15 lettuce heads per m^2^. The teams’ algorithms had to automatically make the spacing decisions.

The following prices per lettuce head were given Class A = 0.50 EUR/head, Class B = 0.40 EUR/head, and Class C = 0.00 EUR/head. In commercial practice, harvested lettuce heads are sold per head, but as in reality, the economics of the greenhouse is eventually expressed in resource usage and production per average m^2^ of the growing area. Therefore, the price of the lettuce was multiplied by the average number of heads per m^2^ of the growing area. The formula to calculate the average lettuce crop density (heads/m^2^) is the following:

(1) $$AverageCropDensity=\frac{D}{\sum_{d=1}^{D}\frac{1}{density_{d}}}$$

where D is the total number of days since transplanting until harvest and $density_{d}$ is the plant density at day d.

Teams had to maximize net profit. Net profit was calculated from income minus costs. Income was determined by multiplying the yield with the price per class. The total costs consisted of fixed and variable components associated with the greenhouse operation. On top of that teams were ‘charged’ for every manual intervention on their autonomous algorithm (EUR 1 per intervention). This penalty was meant to strongly discourage such interventions ensuring that the algorithms would work as autonomously as possible. Fixed costs accounted for the plant material, maintenance, and depreciation costs of the greenhouse equipment. The variable costs accounted for the resource use (electricity for artificial lighting, energy for heating, and CO_2_ injection).

### 2.4. Data Communication

**Figure 3:**
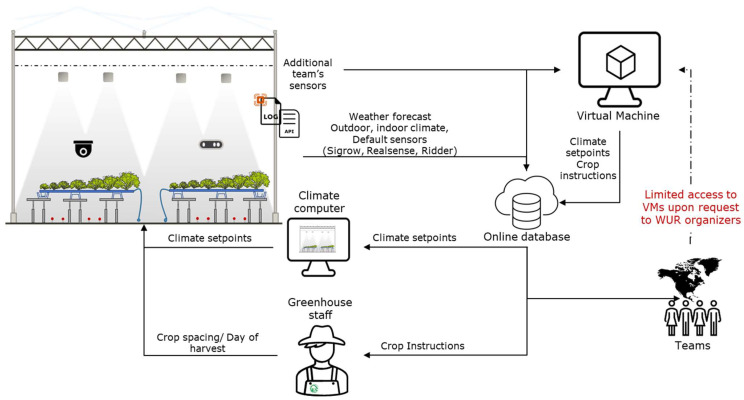
Data communication structure Data flows from indoor and outdoor climate and additional sensors to the virtual machines and the online database. Decisions of algorithms of teams are written from the Virtual Machines to the online database from where another communication protocol writes the controls to the greenhouse climate computer before their implementation in the actual greenhouse compartments. Greenhouse staff receives decisions from online database for spacing and moment of harvest.

Data communication between the underlying systems was vital to ensure a stable, uninterrupted integration and operation. In this experiment, an Azure file share was made available to ensure enough storage capacity for collected datasets. Azure Virtual Machines—NCasT4_v3-series (VMs) were used for high-performance computing and deploying AI workloads, such as real-time inferencing of user requests. The infrastructure supported the communication between the greenhouse climate computer, control systems, sensing devices, and the state of actuators, measured indoor and outdoor climate (Figure 3) (Appendix A Table A1). Numerical time-series data of the realized controls, climate, and additional sensor sensors can be found under the Data Availability Statement.

### 2.5. Remote Sensing and Data Collection

In each greenhouse compartment, standard sensors were made available, comparable to earlier experiments described in [21,22]. These consist of an outside weather station, obtained weather forecast, and indoor climate parameters (temperature, relative air humidity, PAR light, CO_2_) along with the status of all actuators (heating, fogging, lighting, screening, CO_2_) in 5 min intervals. The output of the standard sensors was continuously available as input for the teams’ algorithms.

In commercial production, lettuce traits are seldom collected during the growing cycle and crop performance is evaluated by growers’ visual inspections only. In this experiment, RealSense D415 [57] cameras were hung 1 m above the growing crop in the area of evaluation. The camera uses stereo vision and stores depth, RGB, and IR images. All camera parameters, both intrinsic and extrinsic, are provided with the published dataset under the Data Availability Statement. These parameters could be used to convert the images to point clouds. Images were taken every 15 min in each compartment during the cultivation cycles.

**Figure 4:**
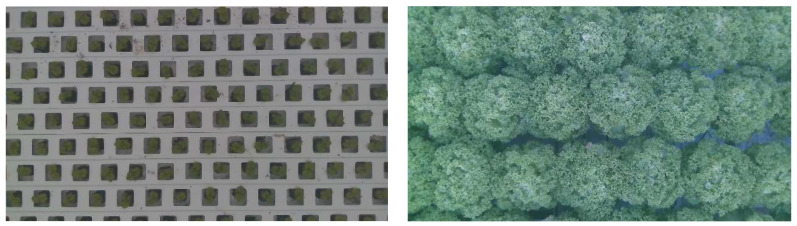
Two example images of RealSense D415 from the day of planting (**left**) and the day of harvest (**right**). On the day of harvest, plants were sampled from the field of view of the camera and were destructively measured for height, diameter, fresh weight, dry weight, and quality.

Periodic destructive harvests of six plants per compartment were taken on the day of planting and subsequently on a weekly basis Destructive measurements of plant height, diameter, fresh weight, and dry weight, and scores for leaf deformation due to outer leaf tip burn were carried out. The individual lettuce plants were taken from the right and left side of each compartment as shown in Figure 2b. Next to that, images of the individual plants were made each 15 min; an example can be found in (Figure 4).

### 2.6. Image Processing for Plant Spacing Decisions

One of the main research questions is how images taken in a greenhouse conform to commercial practice and computer vision can be used to determine the optimal spacing strategy. To do this, the images of the lettuce crop in the greenhouse need to be related to a relevant crop variable, such as crop growth rate. From the time series of images taken inside the greenhouses (Figure 4), the coverage can be calculated over time. The coverage can be defined as the area covered with green leaves relative to the total ground surface area. However, coverage might not be a good indicator to determine plant growth rate, as plant growth rate may decline once the leaves touch neighboring plants. It can be assumed that for very high coverage growth is hampered. So, coverage might not be a suitable parameter to be used for spacing decisions. Crop volume on the contrary might describe plant growth rate even if the coverage is close to 100%. Crop volume can be estimated by coverage and height and can be used to determine crop growth rates in time. The volume over time is a relevant crop parameter, however, it might not directly assess if spacing was carried out correctly. Another option might be to calculate light interception (or light loss). The different methods of “coverage” (Section 2.6.2), “volume over time” (Section 2.6.3), and “light loss over time” (Section 2.6.4) are explained in the following sections after describing how crop segmentation (Section 2.6.1) from greenhouse images is implemented.

For optimizing spacing decisions, the challenge lies in realizing a fast plant growth rate on one hand and limited use of space on the other hand. Early spacing facilitates fast growth rates thus increasing yield over time, late spacing facilitates less occupation of space thus decreasing resource use. An optimum spacing decision is therefore necessary.

#### 2.6.1. Crop Segmentation

**Figure 5:**
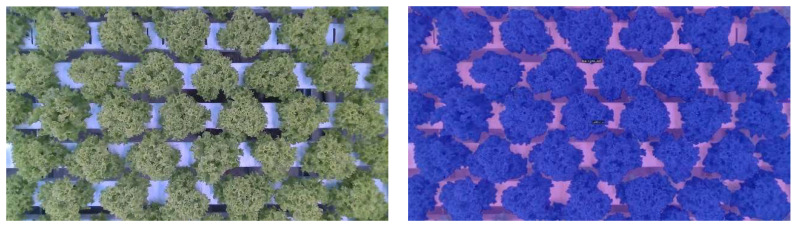
Example of original (**left**) and annotated image (**right**). The annotated image has two classes in blue the lettuce class and pink the background class.

First, the lettuce crop needs to be segmented from the background. The growth of each lettuce head over time was identified using instance segmentation. However, this is only possible in the first 2 weeks. After that, the lettuce heads start to touch each other, and leaves overlapped. As visible in Figure 5 it is almost impossible to identify which leaves belong to which lettuce head. Therefore, semantic segmentation is used in this study. Specifically, DeepLabv3+, which is implemented in detectron2 (v0.6) [58]. For training purposes 23 images have been annotated, in which each pixel was either annotated as background or lettuce, an example is shown in Figure 5. All settings for training were kept the same as in the original implementation detectron2. Only the number of iterations was set to 2500 and the image size was set to 1024 × 1024. For validation purposes, 12 additional images have been annotated. The evaluation was carried out on the validation dataset using the mean Intersection over Union (mIoU) metric Equation (2). In which M denotes the mask of the class, respectively.

(2) $$mIoU=\frac{\left(\left|\frac{M_{GTlettuce}\;\cap \;M_{Lettuce}}{M_{GTlettuce}\;\cup \;M_{Lettuce}}\right|+\left|\frac{M_{GTbackground}\;\cap \;M_{background}}{M_{GTbackground}\;\cup \;M_{background}}\right|\right)}{2}$$

#### 2.6.2. Coverage

The coverage was calculated by segmenting the images with the trained DeepLabv3+ model. In these segmented images the number of pixels classified as lettuce was divided by the total number of pixels, see Equation (3).

(3) $$Coverage\left[\%\right]=\frac{\#\;lettuce\;pixels\;}{\#\;total\;pixels}\cdot 100$$

#### 2.6.3. Volume over Time

**Figure 6:**
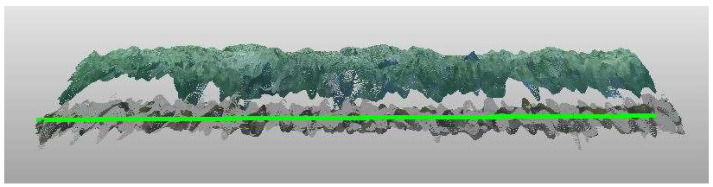
Side view of point cloud within green the fitted ground plane using RANSAC [58] to determine the height. At the bottom the plants at the start date and above the plants at the day of harvest.

To determine the lettuce head volume over time from images taken in the greenhouse with conditions that conform to practice a “ground plane” is needed. This ground plane was determined by fitting a plane using RANSAC [59] through the non-lettuce pixels on the day of planting. RANSAC can compensate for slight skewness in camera mounting. This method assumes that the camera position does not change after planting. The height is subsequently calculated by determining the point-to-plane distance for each pixel classified as lettuce (Figure 6). The volume was then calculated by multiplying the height by the pixel size in mm and dividing by the density at each moment in time. By dividing by the density, the volume per plant was calculated which was needed to correct for different plant densities. The growth was then determined based on the volume increase.

(4) $$Height_{i}\left[\mathrm{cm}\right]\;=\;dist\_plane_{i}-dist_{i}$$

(5) $$Volume\;over\;time\;\left[{\mathrm{cm}}^{3}\right]\;=\;\frac{\sum_{i=0}^{N}\left(Height_{i}\cdot pixelsize^{2}\right)}{density}\cdot 1000$$

#### 2.6.4. Light Loss over Time

For optimal use of the greenhouse area, it is important to evaluate the spacing decision. In this research, the light loss was calculated over time. Simplified we determined if the light loss after spacing was larger than the light loss before spacing due to overlapping leaves. The light loss after spacing was calculated by subtracting 100 minus the current coverage (Section 2.6.2) (Equation (6)). The light loss before spacing was calculated by determining the difference between the current coverage and the theoretical coverage. This theoretical coverage was the projection of previous coverage divided by the previous density and multiplied by the new density (Equation (7)). This difference indicated how much light was lost due to overlapping leaves. Now the light loss was calculated (Equation (8)). If for example, the light loss was negative, it indicated that too many leaves were overlapping, resulting in a light loss that was larger than the light loss after spacing. On the other hand, if the light loss was positive, then the spacing decision was too early, because in the new spacing density, there was more light lost than before.

(6) $$LightLoss_{current}=100-coverage_{t}$$

(7) $$LightLoss_{before\;spacing}=coverage_{t}-\frac{coverage_{t-1}}{density_{t-1}}density_{t}$$

(8) $$LightLoss=LightLoss_{current}-LightLoss_{before\;spacing}\;$$

where t denotes the time when spacing occurred and t−1 time before spacing.

## 3. Results

Two experiments were carried out consecutively. The first experiment offered the teams the possibility to test their algorithms in growing a real crop in a real greenhouse to bridge the gap between simulation and reality. The second experiment was the eventual challenge that determined the winner of this third Autonomous Greenhouse Challenge. In both experiments, the algorithms were optimizing income against costs to achieve a maximum net profit. The challenge was the outside conditions during the second experiment (early summer) were very different from those in the first experiment (late winter). Both data sets are made publicly available and can be used for further development of intelligent control of lettuce production systems. We show both but focus more on the second data set which was determining the winner of the competition.

The results of realized climate, resource use, crop yield, and applied plant spacing are given for the two cultivation cycles in the six greenhouse compartments.

### 3.1. Climate and Resource Use Analysis

**Figure 7:**
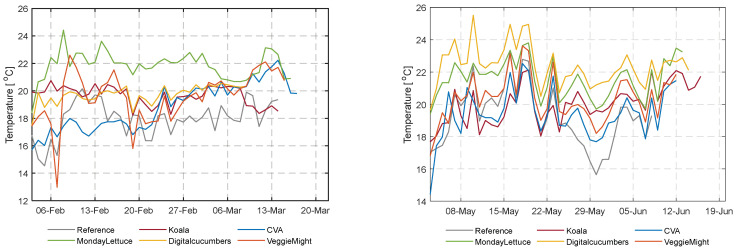
Average daily temperature (°C) of all compartments during the first and second cultivation.

**Figure 8:**
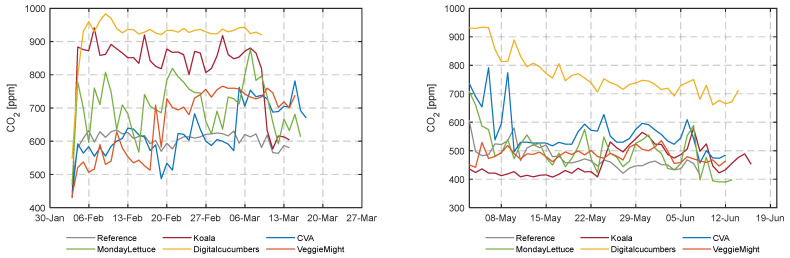
CO_2_ concentration (ppm) for the different compartments during the first and second cultivation.

**Figure A1:**
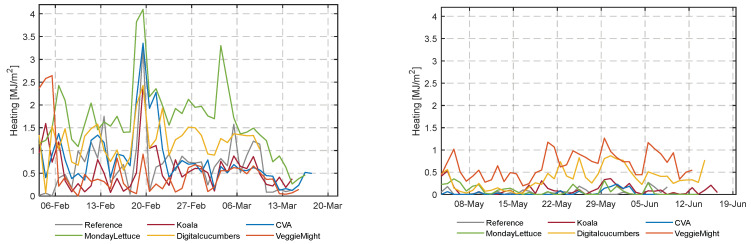
Heating energy consumption (MJ/m^2^) of all compartments during the first and second cultivation.

The climate control strategy in the greenhouse largely determines the use of resources. Figure 7 illustrates the average daily greenhouse air temperature in the different compartments during the late winter and early summer experiments. The realized daily average temperature ranged between 18 °C and 22.5 °C for the first experiment (winter), whereas for the second experiment (early summer) the minimum and maximum diurnal temperatures were on average 1 °C higher. Teams decreased their energy consumption for heating by more than 80%, up to 97%, except for two teams DigitalCucumbers and VeggieMight (Appendix A Figure A1). For DigitalCucumbers the operation of the heating pipes is reflected in the high diurnal temperature realized in their compartment augmented by their low ventilation rates that maintained the highest CO_2_ concentration (Figure 8), despite the low CO_2_ dosage rates. VeggieMight realized lower temperatures, due to the higher ventilation rates, despite the higher energy used for heating. Appendix A Figure A1 shows the energy consumption for heating over time in both experiments, an important part of the resource use.

**Figure 9:**
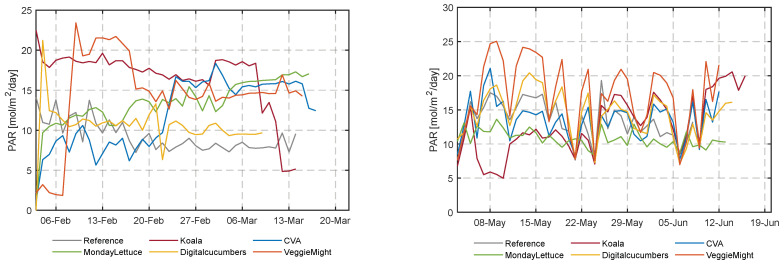
Indoor photosynthetic active radiation (PAR) for the different compartments during the first and second cultivation.

**Figure 10:**
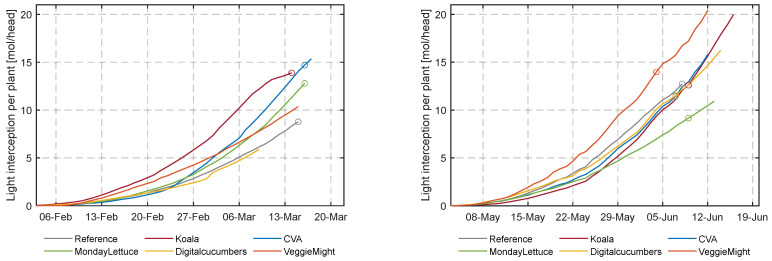
Cumulative light intercepted by each lettuce head per compartment in the first and second cultivation. Light interception per lettuce head was calculated by a multiplication of the daily light integral (DLI) with the green coverage per m^2^ growing area, divided by the head density on each particular day. The circles (o), mark the days at which the lettuce heads reached the target fresh weight of 250 g by linearly interpolating the data of the weekly destructive measurements on randomly selected lettuce heads.

**Figure A2:**
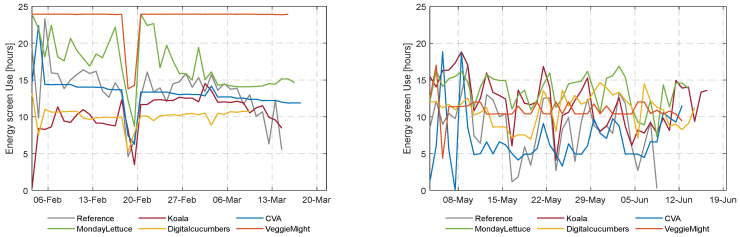
Energy screen usage in all compartments during the first and second cultivation.

**Figure A3:**
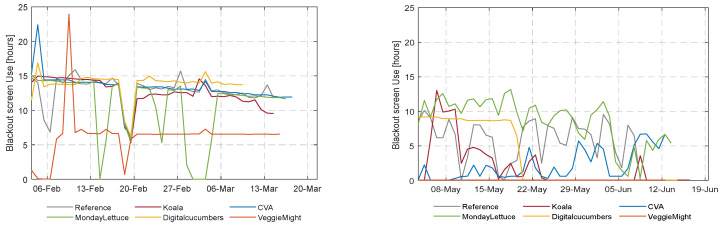
Blackout screen usage in all compartments during the first and second cultivation.

**Figure A4:**
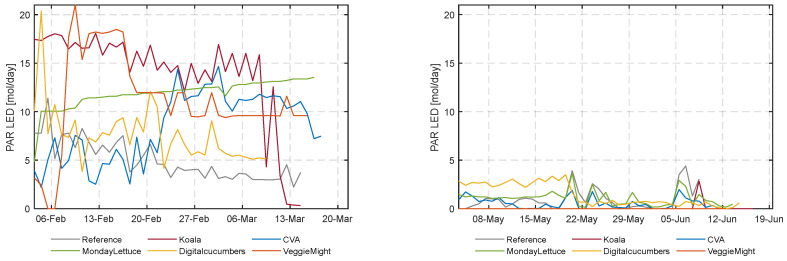
Artificial lighting usage in all compartments during the first and second cultivation.

**Figure A5:**
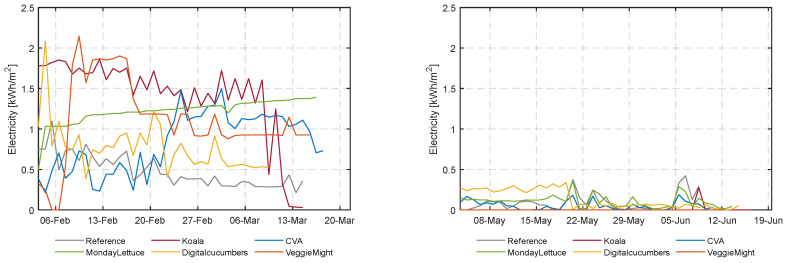
Electricity consumption (kWh/m^2^) in all compartments during the first and second cultivation.

The daily light integral (DLI), mol m^−2^ plant^−1^ in the greenhouse compartments is the sum of outside sunlight, influenced by the team’s screen usage (Appendix A Figure A2) and topped up with the artificial light for each team (Appendix A Figure A4). Especially the longer day length and higher intensities of solar radiation resulted in a higher cumulative DLI during the second experiment. The realized indoor daily PAR for each team as the sum of solar radiation and artificial lightduring the two cultivation experiments is illustrated in Figure 9. In the second experiment, team VeggieMight realized the highest cumulative DLI (Figure 10) despite the zero hours of their artificial illumination (Appendix A Figure A4) and the highest total light interception per head of lettuce, as a result of the intelligent operation of their blackout screen (Appendix A Figure A3), and their low plant density (Table 1). Comparable cumulative DLIs for all teams were observed in the first experiment. However, in the first experiment, the light demand of lettuce was mainly covered with artificial light, as 50% to 88% of the measured light originated from LEDs (Appendix A Figure A4). During the second experiment, comparable light levels were again required to reach the target weight, illustrated by the colored circles in both figures. Due to the higher solar radiation, less artificial light was needed in this experiment. Appendix A Figure A5 shows the electricity consumption over time in both experiments of each team, an important part of the resource use.

All climate strategies applied by the teams resulted in differences in resource use which are summarized in Table 2.

### 3.2. Crop Yield Analysis

**Figure 11:**
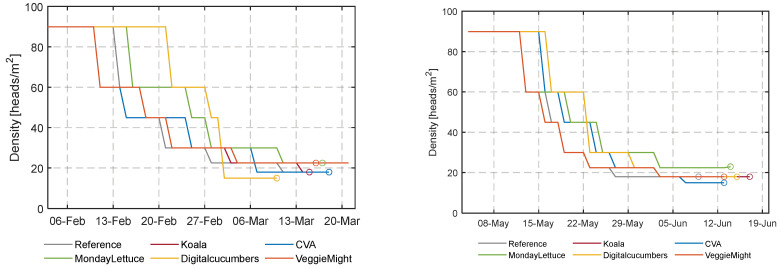
Lettuce density (heads m^2^) and harvest dates (o) in the different compartments during the first and second cultivation period.

Figure 11 shows the initial plant densities of 92 heads/m^2^ chosen by all teams and the different densities realized over time. Throughout the cultivation periods, lettuce heads were spaced according to the decisions of each team’s algorithm. The reason for the spacing was to balance fast crop growth and minimize greenhouse space utilization thus resource use and costs for spacing. As the average plant density impacted costs due to resource use and labor for spacing events, teams MondayLettuce and DigitalCucumbers attempted to exploit the advantage of higher densities and fewer spacing interventions, respectively. The average plant densities of the two cultivation cycles for the six compartments are shown in Table 1.

**Figure 12:**
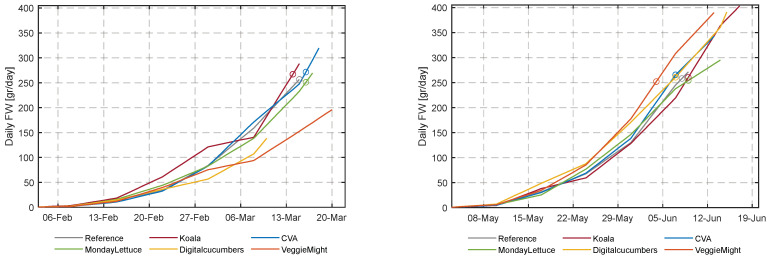
Development of fresh weight [g/plant] in the different compartments for the first and second cultivation. The curves were obtained by linear interpolation of the weekly randomly sampled plants and (destructively) weighed heads of lettuce. The end of the lines represents the chosen date of harvest and the fresh weight at harvest. The circles (o), represent the days on which the lettuce heads reached the target fresh weight of 250 g.

During the first experiment, the team’s algorithms seemed to have computed the harvest time quite accurately at the target weight of 250 g, as can be seen in Figure 12. The crops of DigitalCucumbers and VeggieMight grew poorly, they were still far off from the targeted weight the moment that the first cultivation was terminated. For the second experiment, the algorithms of all participants were too late in harvesting, the harvest weight was higher than the target weight. Only the reference compartment, was harvested timely. Appendix A Table A3 summarizes the lettuce weight at harvest and the number of cultivation days per compartment. It also shows the dates at which the ideal target weight would have been achieved for the different compartments by linearly interpolating the weekly fresh weight measurement.

**Figure A6:**
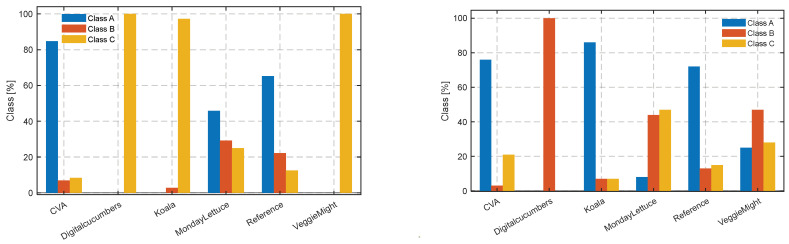
Classification of lettuces at the different harvest moments as Class A, Class B, and Class C lettuces for the first (**left**) and second (**right**) cultivation. The classification was carried out using a standardized protocol that distinguished the harvested products given the satisfaction of the target fresh weight, the presence, and severity of the outer-leaf burn, and the presence of diseases and or malformations that resulted in non-sellable products.

Total lettuce crop yields (Figure 12 and Appendix A Table A3 and the quality assessment (Appendix A Figure A6) resulted in a computed income from this cultivation cycle. In Section 3.3 this income is compared with the costs associated with the second cultivation cycle to obtain the computed net profit (Table 2).

### 3.3. Net Profit

The combination of climate strategies, resource use, crop yield, and quality realized by the teams resulted in different net profits. Details are shown in Table 2.

### 3.4. Plant Spacing Analysis

The net profit is relying on crop yield, quality, resource use, and greenhouse occupation. The realized plant growth rates, plant densities, and realized final harvest due to timely estimation of plant weights were shown to be crucial for the net profit. Therefore, the options of different detailed computer vision analyses to make timely decisions on spacing decisions are shown in this paper.

#### 3.4.1. Coverage

**Figure 13:**
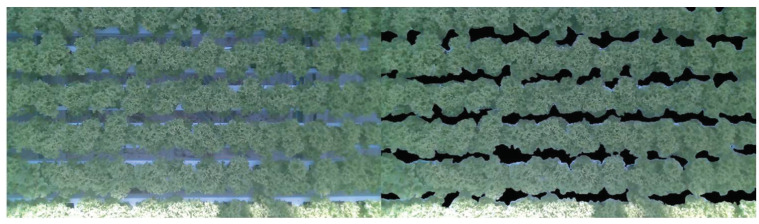
Example of a real (**left**) and segmented (**right**) image using DeepLabv3+. From the segmentation, the coverage [%] was calculated.

The computer-vision-based data analysis of plant growth mainly relies on the segmentation of the images of lettuces taken at a defined area over time. An example of such an image is shown in Figure 12 (left). There results of being segmented with the DeepLabv3+ algorithm as described in the Materials and Methods sectionare shown in Figure 13 (right). The algorithm had a mIoU of 98.2% on the validation dataset, with 100% indicating that the segmentation is perfect. Even though the validation dataset was relatively small, the segmentation procedure can be considered to be sufficiently robust, since only a small fraction of processed images from the datasets segmentation occurred to be incorrect. An example is given in Figure 13. Although there are white edges visible (right), showing where pixels were falsely assigned as ‘lettuce’, these edges are relatively small. The algorithm appeared also to be robust in dealing with different light conditions in the greenhouses as shown in the correctly processed bottom row of Figure 13.

**Figure 14:**
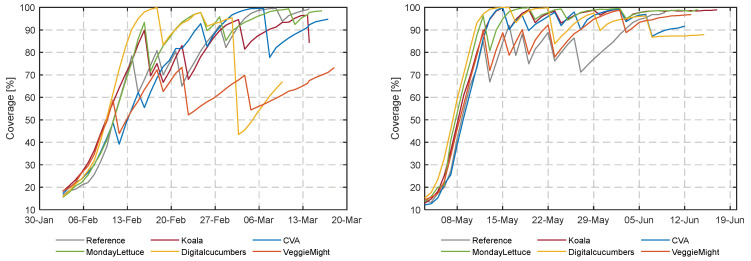
Coverage [%] of the greenhouse with lettuce heads, as calculated with the segmentation algorithm The frequently shown stepwise fall of coverage is a result of spacing actions.

Figure 14 shows the percentage of cultivation area covered by lettuce for each image over time for each compartment. In the first experiment, less efficient space occupation and coverage were observed, due to the explorative decision-making of the teams. Considering the more strategic decisions during the second experiment, teams targeted more efficient space occupation. A high coverage percentage was realized in a shorter time. Most teams maintained a coverage above 90%, only teams VeggieMight and Reference seemed to have spaced too early if only coverage is considered for the decision making. However, as explained in the materials and methods section, coverage might not be a good parameter to decide on spacing, since growth might be hampered already by too late spacing.

#### 3.4.2. Crop Volume over Time

**Figure 15:**
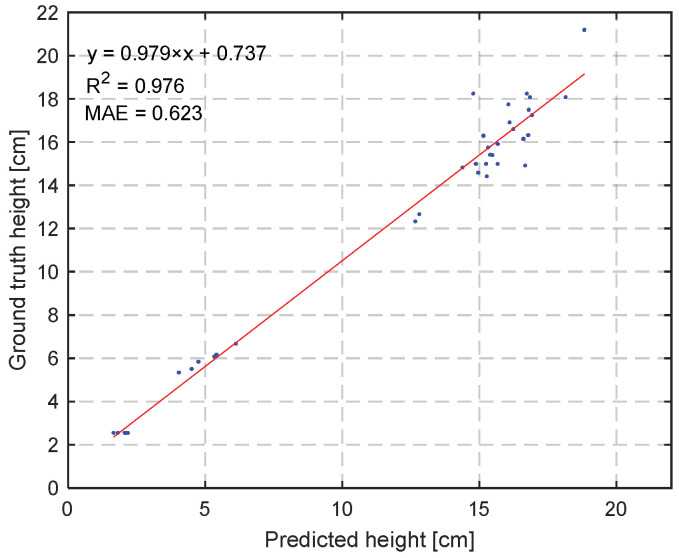
Correlation of manually measured ground truth height (ground truth height) of lettuces during destructive measurements compared with predicted height (predicted height) from RealSense camera images of lettuces.

Next to coverage, crop width, height, and volume are suitable crop traits associated with growth. To explore the potential of these traits, in this research height and volume were determined over time. Since volume is strongly correlated with height, a comparison between calculated and manual crop height is shown in Figure 15. Figure 15 shows a strong correlation between the calculated height as it follows from the RealSense camera images and the manually measured height (ground truth), with a high R^2^ and slope close to 1. Therefore, daily height measurements from the RealSense camera were assumed to be correct and were used to calculate the volume.

**Figure 16:**
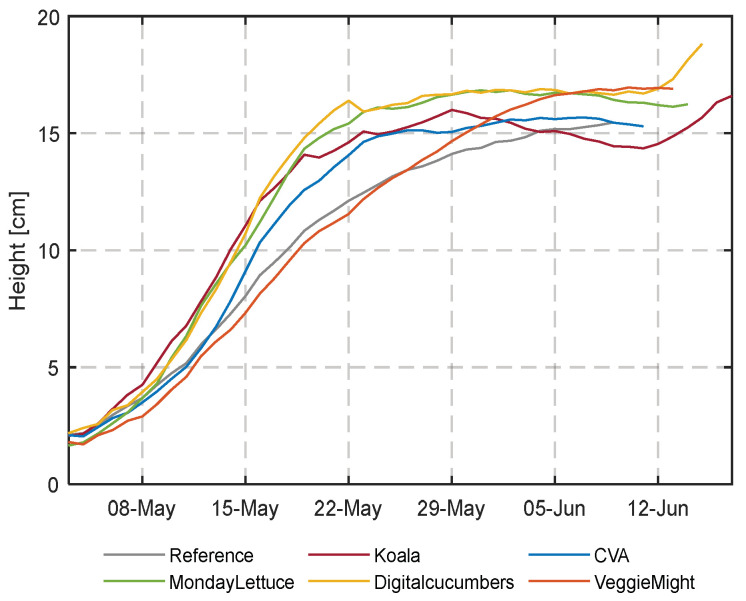
Daily calculated lettuce height from RealSense camera images in all compartments during the cultivation start.

**Figure 17:**
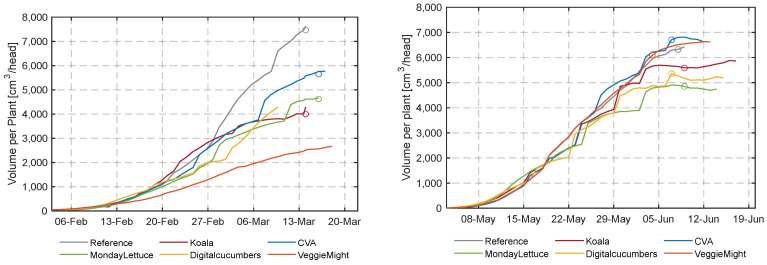
Daily estimated lettuce volume in all compartments during the cultivation period.

In Figure 16, the calculated height from images taken by the RealSense camera over time is shown for all compartments. Reference and VeggieMight have a lower predicted plant height due to the early spacing decision. DigitalCucumbers has the highest plant height, elongation occurred when plants were touching each other due to high density. An interesting phenomenon is that after the last spacing decision (ca. after 5 June) not only height (Figure 16) but also volume (Figure 17) is reaching a plateau. This means that the daily fresh weight increase (Figure 12) of the last weeks is not visible in this method of image analysis.

In Figure 17 the daily estimated plant volume is given using imaging. The circles indicate on which day the target harvest weight of 250 g was reached. Large differences between the compartments were observed. In the second experiment, the difference in volume between CVA and MondayLettuce is remarkable. At the optimal harvest day, CVA has a volume of 6705 cm^3^/head and MondayLettuce 4844 cm^3^/head. A part of this large difference is caused by the plant density, which is at the end 15 and 22.5 plants/m^2^ for CVA and MondayLettuce, respectively. Because of the high plant density of MondayLettuce, more leaves were overlapping (coverage was 98.5%, compared to 87.2% of CVA). Since overlapping leaves do not contribute to volume in the image analysis it explains the lower volume of MondayLettuce with respect to CVA (Figure 17). This phenomenon is also summarized in Table 3. In this table, the volume is sorted from high to low. Although the weight of the lettuce at optimal harvest day in each compartment is approximately 250 g, there are differences in volume per head for similar crop densities. The Reference for example had a much higher volume than Koala. Both Figure 16 and Figure 17, and Table 3 show differences from the similar weight. From this, it can be concluded that the volume calculation alone will not be a conclusive trait for the estimation of the weight of the head of lettuce.

#### 3.4.3. Harvest Indicator over Time

As presented in the previous section teams could have harvested earlier given the target harvest weight of 250 g per head. From Figure 17 and Table 3, there were differences in volume for similar harvest moments, indicating that volume might not be an ideal indicator for determining the ideal moment of harvest. The correlation coefficient of all calculated traits from the image analysis can be found in Appendix A Table A3. From this table the area per plant multiplied by the maximum height has the highest correlation coefficient, higher than volume.

**Figure 18:**
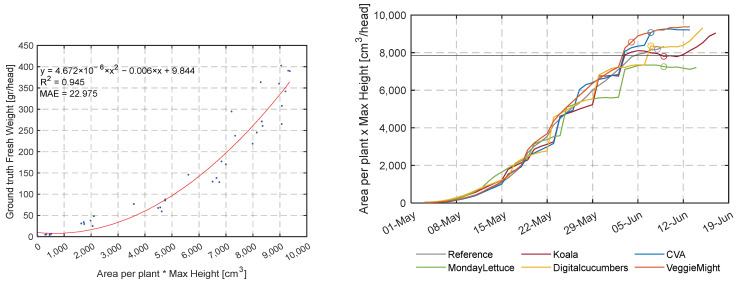
Relation of calculated area per plant multiplied by maximum height [cm^3^] and measured fresh weight [g/head] (**left**). Area per plant multiplied by maximum height [cm^3^/head] as a harvest indicator in realizing the target weight of 250 g/head in all compartments (dots) (**right**).

In Figure 18 (left), the fresh weight as a function of area per plant multiplied by maximum height is visualized. From this figure, it can be concluded that there is still some noise, the Mean Absolute Error (MAE) is 22.98 g/head and RMSE of 31.2. According to the second-order equation, the harvest weight is reached at 7840 cm^3^.

Figure 18 (right) illustrates the area per plant multiplied by the maximum height as the most representative harvest indicator. The colored circles indicated the moments of harvest that satisfy the fresh weight criterion 250 g whereas the grey horizontal line depicts the moment that the harvest indicator of 7840 cm^3^ is satisfied. In Table 4 the exact dates of the fresh weight criterion and harvest indicator are given for the different teams.

#### 3.4.4. Light Loss over Time

**Figure 19:**
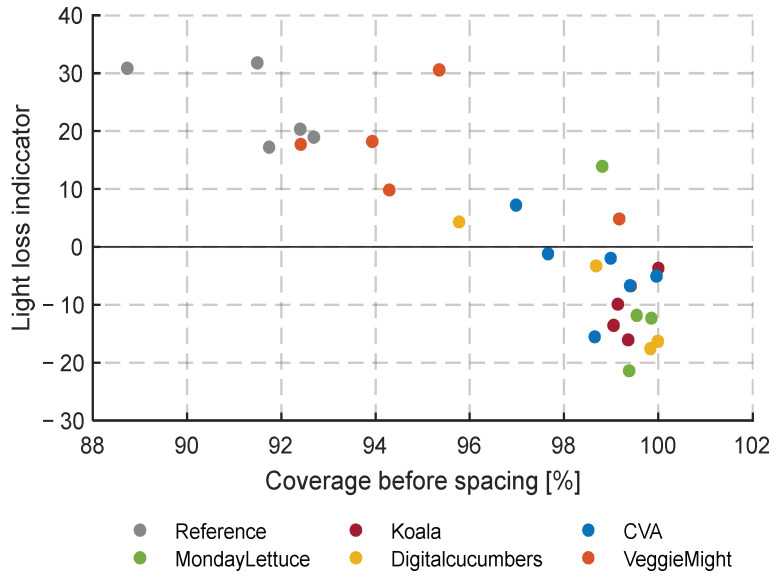
Light loss calculation for all compartments for the spacing instances of the second experiment. Negative values indicate a (too) late spacing and large positive values a too-early spacing.

In Figure 19, the result of the calculation of light loss over time of the second experiment is shown. This light loss factor can be calculated by comparing the coverage factor just before and just after a spacing instance. Therefore, the result yields a number of points rather than a time series. The hypothesis is that spacing is optimal when the light loss calculation gives a result close to zero at each spacing action. A light loss calculation close to zero means that the lettuce heads just started touching each other by the time that the spacing was performed. This allows for minimal greenhouse space occupation, which saves on resources, whereas quality losses are prevented. Figure 19 shows that especially VeggieMight and the Reference spaced too early, whereas Koala always spaced very late. DigitalCucumbers had two good spacing moments at the end of the experiment but was too late for two others. In the beginning, for both the first and second spacing decisions, they were the latest team which resulted in large light losses and irreversible damage to the crop (Appendix A Figure A6). CVA seemed to have the best spacing strategy since most of their spacing decisions were made with light loss points close to zero. However, even this team had once a large light loss smaller than −10. Keep in mind that the calculation of light loss can only be carried out after spacing. It should therefore be treated as an observable parameter to train decision-making algorithms that base the decision on (a combination of) covered fraction and average head volume.

After combining Figure 19 with Figure 14, we learn that 98% seems to be a reasonable coverage strategy for autonomous spacing decisions.

## 4. Discussion

In the experiment of this study, the strategic and operational scheme of lettuce crop cultivation was determined by AI algorithms developed by teams participating in the challenge. These AI algorithms were based on greenhouse climate and crop sensor information. The final optimization target was net profit, thus on the one hand side a high crop growth rate and high plant quality for a high income and on the other hand low resource use for low costs. Since greenhouse occupation is essential, optimal plant spacing decisions are important.

Commercial lettuce growing is a continuous process of daily planting young plantlets and harvesting the full-grown lettuce heads. Target weight is realized over a reasonable time window (6–8 weeks) dependent on the cultivation strategy. Economics were expressed per m^2^ of the production area, therefore the resource use and selling prices were multiplied by the average number of lettuce heads per m^2^ (Equation (2)).

Teams had two cultivation cycles. The first cycle was used by the teams to test and explore their algorithms, the second cycle determined the winner. As this means that the teams must have applied their latest skills and knowledge in this second growing cycle, the discussion is focusing on the early summer results.

For an efficient greenhouse occupation, and to leverage the effect of the average density of lettuce heads on the final profit, some teams maintained high densities (Table 1). At high densities, neighboring plants competed for light (Figure 10). In both experimental cycles, 11–15 mols PAR/head was needed to realize the target weight of 250 g per head. However, MondayLettuce used only 9 mol/head in the second experiment. This team maintained a low cumulative DLI and in combination with the highest density among all teams in the second cultivation, it yielded the lowest amount of total light interception per plant. Also, DigitalCucumbers realized a high density. The high plant density resulted in intertwined root systems that made the first spacing difficult and seems to be linked to the outer tip burn (Appendix A Figure A6) and the aversively malformed and elongated plants (Figure 16).

Team VeggieMight realized the highest cumulative indoor PAR, even without applying any supplemental lighting (Appendix A Figure A4). This was a result of zero deployment hours of the blackout screen and a very limited deployment of the energy screenThe choice not to use any lighting or any blackout screens saved fixed costs associated with the equipment and the associated running costs for electricity. However, also this team suffered from the occurrence of outer tip burn and malformations on the plants, even though they had the lowest average plant density. The high fraction of class C products resulted in a low income. Similar to VeggieMight, team Koala, did not use supplemental lighting. This team was also restrictive with CO_2_ dosing in the first weeks of the cropping cycle. The team maintained a high coverage bouncing from 93% to 98.9%. The fact that the algorithm of this team managed to reduce costs, managed to have a high average head density and had a high fraction of class A resulted in team Koala being the winner.

The final harvest was too late for all teams (Figure 12). Timely harvest would have resulted in lower resource use and higher average plant density. The effect of earlier harvest on net profit cannot be quantified, unfortunately, since the quality of the lettuce heads at earlier moments in time cannot be predicted from the collected data.

Contrary to commercial greenhouse operations with continuous planting, spacing, and harvesting, the two growing cycles of this study concerned single batches. The choice for single batches was required to fit the format of the Autonomous Greenhouse Challenge aimed at allowing teams to develop and show the potential of autonomous algorithms growing a crop based on data analyses and vision. As a result, the computed profits, although valid according to the rules of the Challenge, cannot completely be compared with commercial practice. Dedicated trials would be needed to reflect deeper on the lettuce growth responses in continuous commercial cycles. However, such trials were outside the scope of this research. Nevertheless, results show that greenhouse occupation is essential and that optimum plant spacing decisions are important.

In fully autonomous cultivation such decisions should be made based on continuous sensor information. In this study camera images obtained by RealSense cameras in the greenhouse were used to obtain information on crop growth. DeepLabv3+ was used to separate the lettuce from the background. The model was only trained with a minimal amount of data. However, considering the output images in 3.4 and the high mIoU (98.2) it can be concluded that the segmentation proved suitable to be used as a base for crop spacing decisions.

The RealSense cameras also provided data on the development of height and volume over time. We expected that these two traits could be used to describe growth. The development of volume over time has been related to biomass, as in [60,61]. As the height and width information was proved to be very accurate (a mIoU of 98.2% for the covering fraction and an R^2^ of 0.976 for the height estimation) the lettuce head volume could be reasonably estimated. However, the computed volume showed to be not suitable to predict the crop weight. First, this can be explained by the fact that overlapping leaves do not contribute to coverage or volume. Secondly, in Figure 16 the height over time flattens during the last 2 weeks, and related to that, in Figure 18 also the volume flattens during the last days. At the same time, destructive measurements show that the fresh weight grows especially in these last days. As neither the coverage nor the height and volume indicated this fresh weight growth, it can be concluded that in the final stage, growth takes place from the central point of the head, resulting in more compact lettuce heads.

The product of the multiplied area per lettuce head with the maximum height resulted in the highest correlation coefficient with fresh weight (Appendix A Table A3). Three papers using the [50,51,52] dataset had a RMSE up to 25.3. As indicated, we obtained a lower accuracy, however, we should take into account that the datasets are not fully comparable. Our dataset is made within the greenhouse, with many plants and resultingly overlapping leaves. The previous dataset and other research in lettuce growth contained data of single plants only [53,55]. In our research, the predicted fresh weight was able to determine non-destructively the moment of harvest for the majority of the teams. The suggested harvest indicator dates can be closely related to the harvest dates that satisfy the target weight criterion deducted from the intermediate destructive harvests. For MondayLettuce no results were derived as the high final density of the team resulted in notably lower volume for the team (Table 3) that was hampered by the high leaf occlusion.

The light loss indicator proved to be a good and automatically computable parameter to judge spacing decisions just after the spacing was performed. This hindsight factor is therefore welcome as an indicator to learn about the suitable covering factor to use as a threshold for making a spacing step. In Section 3.4.4, based on the light loss indicator, a covering factor of 98% seemed to be a suitable moment for spacing. The results of teams that spaced at even higher covering factors correlated with more severe issues with outer leaf tip burn and malformations and are therefore not advisable. Spacing at lower thresholds might have given a better quality but would for sure also lead to higher costs per m^2^ due to lower average plant densities. Further experience with spacing on a lower threshold might show that possible higher quality outweighs the additional costs. The Reference and VeggieMight for example had a light loss indicator that was mostly greater than 10. For these teams, a later spacing strategy would likely not have had negative consequences.

In the future, other harvest indicators can be explored by deploying spectral indexes to describe lettuce growth, to address existing shortcomings (overlapping leaves, increased compactness). Spectral indexes can be successfully linked to the leaf area index in greenhouses [62]. Kizil et al. [63] estimated the yield of lettuce plants using spectral indexes. Although their solution only worked for single plants it might be an opportunity to explore further for the purpose of spacing decisions and fresh weight estimation. Also, it is good to point out that in literature growth from non-destructive measurements is mostly determined under ‘ceteris paribus’ conditions, meaning that the environment does not change. In commercial practice and the given dataset, the environment is continually changing due to different climate, light, and spacing strategies. The latter necessitates the utilization of larger datasets than those currently accessible. The acquisition of such datasets combined with the given dataset has the potential to bridge the divide between academic research and industrial production systems in the future.

## 5. Conclusions

- In the experiment described here, teams autonomously were able to control greenhouse lettuce crop production by AI algorithms.
- Autonomous AI algorithms were developed based on greenhouse climate sensor information in time and on crop images maximizing the net profit of lettuce cultivation.
- Realized crop growth and densities due to timely spacing decisions and realized final target harvest due to timely estimation of crop weight have shown to have a large impact on net profit.
- Images from 3D cameras and intelligent computer vision algorithms are helpful to make timely decisions on plant spacing and final harvest decisions.
- Images of the lettuce crop canopy in the greenhouse have to be related to relevant crop parameters to predict crop growth. From the images inside the greenhouses over time, coverage, crop volume, maximum height, and light loss can be calculated to determine the optimum spacing moment. If the light loss is close to zero, an optimum spacing moment was reached, in our experiments that were at a coverage of 98%. The product of area per plant with a maximum height of the plant is a promising indicator for the moment of harvest given a target weight. Deviations from other destructive indicators are highly linked to the results of the crop’s architecture as the impact of leaf occlusion.
- We have shown that computer vision and deep learning algorithms can be used for automated plant spacing decisions toward the autonomous control of greenhouses. The provided open-source dataset contributes to another step in the development of autonomous greenhouses.
- The reality gap between optimum research and commercial production conditions is a crucial aspect to be considered in computer vision applications. Larger datasets need to be acquired to bridge the gap.
- Early pest and disease detection, real-time inclusion of the volatile market prices, robotics in activities of crop handling are among the next steps for higher levels of automation in horticulture (not part of this research).

## Figures and Tables

**Table 1:** The average density of lettuce heads for the two cultivations as calculated using Equation (1).

Experiment Planting Date	Reference	Koala	CVA	Monday Lettuce	Digital Cucumbers	Veggie Might
3 February	32.7	34.5	31.9	41.4	37.7	32.9
3 May	29.0	30.4	29.9	36.7	31.7	28.7

**Table 2:** Net profit of different teams in the second experiment consisting of crop income minus costs (fixed costs, heating costs, electricity costs, CO_2_ costs, and intervention costs).

	CVA	Veggie Might	Digital Cucumbers	Koala	Monday Lettuce	Reference
Total income [€/m^2^]	12.16	10.38	15.84	14.16	11.83	12.12
Fixed costs [€/m^2^]	7.85	6.41	8.50	7.06	9.64	6.59
Heating Costs [€/m^2^]	0.01	0.29	0.16	0.04	0.03	0.02
Electricity costs [€/m^2^]	0.23	0.00	0.46	0.00	0.45	0.34
CO_2_-costs [€/m^2^]	0.60	0.53	0.34	0.11	0.18	0.53
Total operational costs [€/m^2^]	8.69	7.24	9.45	7.24	10.30	7.48
Intervention Costs [€/m^2^]	2.00	1.00	3.00	1.00	2.00	-
Net profit [€/m^2^]	1.47	2.14	3.39	5.93	−0.47	4.64

**Table 3:** Overview of plant traits at optimal harvest date (when target weight of 250 g per lettuce head is reached). Teams are sorted from high to low volume.

Compartment	Optimal Harvest Date	Density [Heads/m^2^]	Coverage [%]	Max Height [cm]	Volume [cm^3^/Plant]
CVA	7 June 2022	15	87.2	15.7	6705
Reference	8 June 2022	18	96.8	15.3	6379
VeggieMight	4 June 2022	18	90.9	16.5	6090
Koala	9 June 2022	18	98.2	14.4	5581
DigitalCucumbers	7 June 2022	18	86.7	16.8	5356
MondayLettuce	9 June 2022	22.5	98.5	16.4	4844

**Table 4:** Overview of area per plant multiplied by max height criterion at optimal harvest date (when harvest indicator is 7840 cm^3^ per lettuce head is reached).

Compartment	Realized Harvest Date [dd/mm]	Harvest Date Satisfying the FW Criterion [dd/mm]	Harvest Date Satisfying the Area per Plant × Max Height Criterion [dd/mm]	Satisfying the Area per Plant × Max Height Criterion [cm^3^]
Reference	9 June	8 June	5 June	79,144
Koala	17 June	9 June	3 June	78,819
CVA	13 June	7 June	3 June	80,717
MondayLettuce	14 June	9 June	-	-
DigitalCucumbers	15 June	7 June	7 June	83,610
VeggieMight	13 June	4 June	3 June	82,410

## Data Availability

The complete dataset of the 3rd Autonomous Greenhouse Challenge: Time-series data on realized climate with annotated crop lettuce-images is published online at https://doi.org/10.4121/21960932.v1, (accessed on 2 January 2023).

## Appendix A

**Table A1:** Data collected throughout the cultivation cycles for all greenhouse compartments on outdoor and indoor greenhouse climate, weather forecast, requested and realized operational controls, weekly destructive plant measurements on which images were annotated, and final harvest plant data. Data are open access [49,56].

Parameter		Unit	Intervals	Description
Measurement	Outdoor temperature	°C	5 min	Meteo
	Outdoor relative humidity	%	5 min	Meteo
	Global radiation	W/m^2^	5 min	Meteo
	Wind speed	m/s	5 min	Meteo
	Wind direction	-	5 min	Meteo
	Rain	[1 rain–0 dry]	5 min	Meteo
	Heat emission- pyrgeometer	W/m^2^	5 min	Meteo
	Absolute humidity content		5 min	Meteo
	Temperature greenhouse	°C	5 min	Indoor climate
	Relative humidity greenhouse	%	5 min	Indoor climate
	CO_2_ concentration greenhouse	ppm	5 min	Indoor climate
	Humidity deficit	g/m^3^	5 min	Indoor climate
	Leeward vent position	% [0–100]	5 min	Indoor climate
	Windward vent position	% [0–100]	5 min	Indoor climate
	Temperature rail pipe	°C	5 min	Indoor climate
	Assimilation lighting (LED)	% [0–100]	5 min	Indoor climate
	Energy screen position	% [0–100]	5 min	Indoor climate
	Blackout screen position	% [0–100]	5 min	Indoor climate
	Cumulative minutes of CO_2_ dosing	minutes	5 min	Indoor climate
	Heating temperature	°C	5 min	Indoor climate
Forecast	Outdoor temperature	°C	5 min	Meteo
	Outdoor relative humidity	%	5 min	Meteo
	Global radiation	W/m^2^	5 min	Meteo
	Wind speed	m/s	5 min	Meteo
	Degree of cloudiness	[1–8]	5 min	Meteo
Control	Ventilation temperature	°C	5 min	Indoor climate
	Lee side min vent position	% [0–100]	5 min	Indoor climate
	Net pipe minimum	°C	5 min	Indoor climate
	Energy screen	% [0–100]	5 min	Indoor climate
	Blackout screen	% [0–100]	5 min	Indoor climate
	CO_2_	Ppm	5 min	Indoor climate
	Humidity deficit	g/m^3^	5 min	Indoor climate
Crop	A class harvest	g	At harvest	>250 g
	B class harvest	g	At harvest	220–250 g
	C class harvest	g	At harvest	<220 g or visible malformations
	Plant density	#/m^2^	Team dependent	92 plants/m^2^ at transplanting
	Day of harvest	Days after transplanting	Once	Team dependent
	Height	cm	Weekly/At harvest	Weekly sampled plants and at harvest day which was team dependent
	Diameter	cm	Weekly/At harvest	Weekly sampled plants and at harvest day which was team dependent
	Fresh Weight	g	Weekly/At harvest	Weekly sampled plants and at harvest day which was team dependent
	Dry Weight	g	Weekly/At harvest	Weekly sampled plants and at harvest day which was team dependent
	Leaf deformation	[1–3]	Weekly/At harvest	Weekly sampled plants and at harvest day which was team dependent. Scoring protocol 1–3, applies to a head of lettuce
	RGB, depth images	-	End of each cultivation	Annotated single crop and canopy images

**Table A2:** Actuators and defaul sensors installed in all the greenhouse compartments during the growing cycles and description of the installed equipment.

Greenhouse Compartments		Description
Equipment	Rail pipe	Max capacity 129 W/m^2^
	Energy screen	LUXOUS 1547 D FR, Ludvig Svensson
	Blackout screen	OBSCURA 9950 FR W, Ludvig Svensson
	LED lights	Dimming 27–270 µmol/m^2^/s with efficiency 2.4 µmol/J, VYPR 2p, Fluence by Osram
	Fogging	330 g/m^2^/h
	CO_2_ supply	Max capacity 15 g/m^2^/h
	Hydroponic gutters (NFT)	Length 3.2 m, 30 plant holes, 11 cm heart-to-heart distance, 10 cm wide, Hortiplan
	Measuring box	Indoor temperature, relative humidity and CO_2_ sensor in ventilated measuring box placed in the middle of the compartment above the growing crop
	PAR sensor	PAR sensor placed above canopy and below LED lights
	RGB, depth camera	Depth Camera D415—Intel RealSense

**Table A3:** Realized harvest dates and fresh weight at the moment of harvest along with the dates at which the weight target was realized by interpolating linearly the weekly destructive measured data.

Compartment	Realized Harvest Date [dd/mm]	Number of Cultivation Days [Days]	Average FW at Realized Harvest [g/Head]	Harvest Date Satisfying the FW Criterion [dd/mm]	Average FW Satisfying the FW Criterion [g/Head]
Reference	9 June	38	271.18	8 June	258.10
Koala	17 June	46	402.81	9 June	260.50
CVA	13 June	42	342.06	7 June	265.35
MondayLettuce	14 June	43	294.96	9 June	254.02
DigitalCucumbers	15 June	44	390.85	7 June	260.61
VeggieMight	13 June	43	389.80	4 June	251.91

**Table A4:** Correlation coefficient between measured and predicted fresh weight using corresponding parameters derived using RGB and depth image.

Parameters	Correlation Coefficient
Coverage percentage	0.5392
Average height [cm]	0.6953
Median height [cm]	0.6946
Max height [cm]	0.7606
Volume [cm^3^]	0.6785
Head density	−0.7912
Volume per plant [cm^3^/head]	0.8975
Area per plant [cm^2^]	0.8987
Mm per pixel	−0.6784
Area per plant divided by volume per plant	−0.4801
Volume per plant divided by area per plant	0.6741
Area per plant multiplied by volume per plant	0.9214
Area per plant divided by mm per pixel	0.9048
Area per plant divided by the maximum height	0.8126
Area per plant divided by median height	0.8400
Area per plant divided by average height	0.8360
Area per plant multiplied by the maximum height	0.9340
Area per plant multiplied by the median height	0.9048
Area per plant multiplied by average height	0.9065
